# Learning Two-View Correspondences and Geometry via Local Neighborhood Correlation

**DOI:** 10.3390/e23081024

**Published:** 2021-08-09

**Authors:** Luanyuan Dai, Xin Liu, Jingtao Wang, Changcai Yang, Riqing Chen

**Affiliations:** College of Computer and Information Science, Fujian Agriculture and Forestry University, Fuzhou 350002, China; 1191193002@fafu.edu.cn (L.D.); 1191193009@fafu.edu.cn (X.L.); 1201193018@fafu.edu.cn (J.W.); riqing.chen@fafu.edu.cn (R.C.)

**Keywords:** feature matching, outlier removal, pose estimation, neighborhood correlation, correspondence

## Abstract

Seeking quality feature correspondences (also known as matches) is a foundational step in computer vision. In our work, a novel and effective network with a stable local constraint, named the Local Neighborhood Correlation Network (LNCNet), is proposed to capture abundant contextual information of each correspondence in the local region, followed by calculating the essential matrix and camera pose estimation. Firstly, the k-Nearest Neighbor (KNN) algorithm is used to divide the local neighborhood roughly. Then, we calculate the local neighborhood correlation matrix (LNC) between the selected correspondence and other correspondences in the local region, which is used to filter outliers to obtain more accurate local neighborhood information. We cluster the filtered information into feature vectors containing richer neighborhood contextual information so that they can be used to more accurately determine the probability of correspondences as inliers. Extensive experiments have demonstrated that our proposed LNCNet performs better than some state-of-the-art networks to accomplish outlier rejection and camera pose estimation tasks in complex outdoor and indoor scenes.

## 1. Introduction

Feature matching is an essential step in varied computer vision tasks. For instance, it is important for image fusion [1], image alignment [2], panoramic stitching [3], image and point registration [4,5], structure from motion [6] and so forth. Feature matching is composed of four key steps, i.e., extracting feature, feature description, building an initial correspondence set and removing false correspondences (also known as outlier rejection). Generally, due to the given matching images usually with large scale variations, occlusions and so on, false correspondences (also known as outliers) in the initial correspondence set are often inevitable. To alleviate this issue, outlier rejection as a post-processing step to improve the ratio of true correspondences (also known as inlier ratio) of the initial correspondence set is necessary and useful. Meanwhile, quality correspondences are the foundation of the essential matrix calculation and camera pose estimation. Therefore, this paper principally focuses on studying outlier rejection.

The traditional outlier rejection methods (such as Random sampling consensus (RANSAC) [7], coherent point drift (CPD) [8], vector field consensus (VFC) [9], locality preserving matching (LPM) [10], grid-based motion statistics (GMS) [11] and so on) are suitable for specific scenarios. However, as the dataset multiplies exponentially and the outlier ratio steeply increases, the performance of traditional methods slumps, and meanwhile, the outlier rejection methods based on deep learning become popular and effective recently.

Some deep learning-based networks [12,13,14,15,16] use an end-to-end approach to select correct correspondences (also known as inliers) and remove outliers. In learning to find good correspondences (LFGC) [12], Moo et al. have introduced a PointNet-like architecture [17] called ResNet Block and Multi-Layer Perceptrons (MLPs) to deal with each match individually. LFGC [12] does well in capturing global contextual information but ignores the local contextual information. To solve this problem, ACNe [13], order-aware network (OANet) [14] and local neighborhood consensus (LFLN) [15] add local information in their networks. In ACNe [13], Sun et al. have proposed Attentive Context Normalization (ACN) to combine local and global attention and normalize the result. In OANet [14], Zhang et al. have adopted the idea of combining local information with global information and introduced a differentiable pooling layer, differentiable unpooling layer and order-aware filtering block. The differentiable pooling layer is used to cluster correspondence information, and the differentiable unpooling layer is utlized to recover from clusters to correspondences. Meanwhile, the order-aware filtering block can enhance the representation ability of feature map while maintaining the order between the input correspondences and the output correspondences. In LFLN [15], Wang et al. have integrated the idea of local neighborhood consistency into the existing network. These works have achieved good results. However, all of them treat each correspondence indiscriminately, and it does not fit the real scenarios. Of note, the spatial-channel self-attention network (SCSA) [16] adds a spatial-channel self-attention block to focus on potential correct matches and capture abundant contextual information in the global region. However, it overlooks the local geometric relationship between matching pairs.

**Figure 1:**
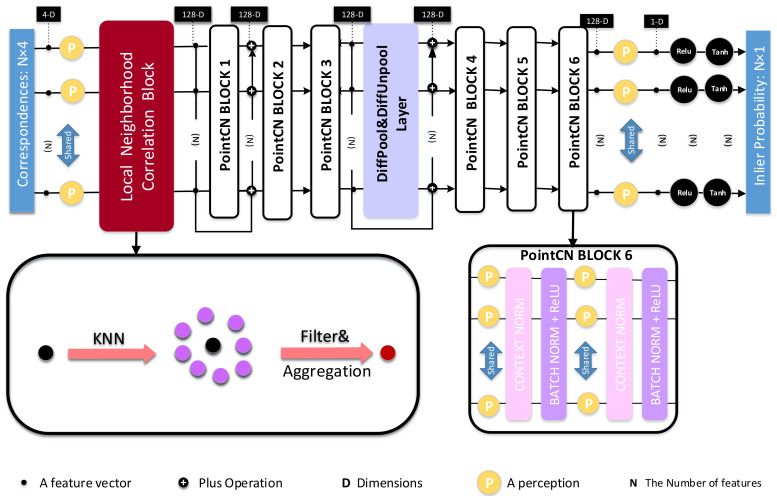
The structure of LNCNet. The DiffPool&DiffUnpool Layer is inserted in the middle of 6 PointCN Blocks, and the processed data are fed into the Local Neighborhood Correlation Block.

There is no network that treats potential inliers and outliers discriminately and considers the local structure at the same time. To figure out the above problem, we present a fresh and useful network, called LNCNet and shown in Figure 1, which can focus on the calculation of potential inliers while considering the local structure. Firstly, we use the k-Nearest Neighbor (KNN) algorithm to loosely determine the local neighborhood. Afterwards, the local neighborhood correlation matrix LNC of the selected correspondence and other correspondences in the local region is calculated, which is used to filter outliers and gain more accurate local neighborhood information. The filtered information is clustered into feature vectors, which contains richer local neighborhood contextual information. After that, we further deal with the above clustered feature vectors. Our LNCNet is capable of determining the probability of each match as a correct match, followed by calculating the essential matrix and camera pose estimation. The comparative and visual experiment results prove that the proposed LNCNet performs better than other comparative algorithms to accomplish outlier rejection and camera pose estimation tasks in the two complex datasets. Our main contributions are summarized:

- In our proposed LNCNet, the local neighborhood correlation block is proposed to filter outliers and cluster more accurate local neighborhood information into new feature vectors.
- In the proposed LNCNet, we construct the local neighborhood from coarse to fine, which can ensure we obtain a trade-off between time and precision.
- Our proposed LNCNet is able to accomplish outlier rejection and camera pose estimation tasks better even under complicated scenes.

## 2. Related Work

Some traditional outlier rejection methods and some deep learning-based outlier rejection networks will be introduced in Section 2.1 and Section 2.2, respectively.

### 2.1. Traditional Outlier Rejection

In feature matching, firstly, a putative match set has been built by some classic and robust methods, such as scale invariant feature transform (SIFT) [18] and newer SuperPoint [19]. Secondly, due to the putative match set generally with numerous false matches, it is necessary to remove outliers. Outlier rejection methods usually include the traditional method and learning-based method. The former has been divided into resampling-based, non-parametric model-based and relaxed method in the literature [20].

RANSAC [7] is a representative in resampling-based methods, which has employed a hypothesize-and-verify tactic to find out correct matches. After that, some variants based on the RANSAC [7] algorithm has been proposed. In MLESAC [21], Torr et al. utilize a maximum likelihood fashion to verify and promote the evaluating indicators. The idea of this work, changing the verification step, has been expanded in the follow-up works. Another pioneering work is locally optimized RANSAC (LO-RANSAC) [22], in which Chum et al. have added a local optimized strategy in the existing well-known models. Recently, Barath et al. have proposed a series of works based on RANSAC [7], including Progressive NAPSAC [23], MAGSAC [24] and MAGSAC++ [25], all of which perform well in the specific scenarios. Though the above methods have been widely used in the computer vision, they fail to cope with image pairs in complex transformations, e.g., non-rigid ones. This condition urges researchers to break away from the resampling paradigm.

A set of non-parametric model-based algorithms have been emerging, which can solve more general prior problems than simple parametric models and can also cope with degraded scenes. In general, these methods model transformations by different deformation functions and utilize different measures to separate outliers and inliers. In recent work, Ma et al. have put forward a new fashion with a $L_{2}E$ estimator to model the transformation and deal with coarse outliers in [26]. Li and Hu utilize the Support Vector Regression strategy to estimate a correspondence function and remove outliers in [27]. In addition, a pioneering work vector field consensus (VFC) [28] has been proposed, during which Ma et al. have proposed a novelty framework to deal with non-rigid matching.

To adapt more complex scenes, relaxed matching methods (geometric constraints become less strict) have been proposed. In LPM [10], Ma et al. have proposed a locality preserving fashion to match, which pays more attention to the local region instead of the global image. It has been proven to be efficient and effective. Meanwhile, in GMS [11], Bian et al. have adopted a simple and effective policy founded on local supporting matches to remove false correspondences. In RFM-SCAN [29], Jiang et al. have projected feature matching to a spatial clustering task with outliers. The purpose is to adaptively gather the initial matches to some motion-consistent clusters as well as an outlier cluster.

Some basic weaknesses are shown in the traditional outlier rejection methods despite being widely applied in computer vision. For example, as the outlier ratio steeply increases in the initial match set, the above traditional algorithms will fail to obtain a good performance. Therefore, deep learning-based outlier rejection arises at the historic moment.

### 2.2. Deep Learning-Based Outlier Rejection

With the exponential increase in the dataset, it becomes popular and useful to utilize the deep learning-based method to deal with points-based tasks. These technologies are approximately divided into parameter fitting [30,31] and point classification and/or segmentation [12,14,32] in the literature [20]. The purpose of the former is to determine the transformation model (i.e., epipolar geometry [33] and fundamental matrix [31]) via the deep learning-based fashion with CNNs. At the same time, the latter prefers training a classifier to distinguish outliers and inliers.

In DSAC [30], Brachmann et al. have substituted probabilistic selection for the deterministic hypothesis selection to reduce the expected loss as well as optimize learning parameters. Afterwards, Ranftl and Koltun have transformed the fundamental matrix estimation into a set of weighted homogeneous least-squares problems, in which the weights are calculated by a deep learning-based network in DFE [31]. In NG-RANSAC [33], Brachmann and Rother have introduced the idea of guiding. Meanwhile, Kluger et al. have added the idea of multiple parametric model fitting in CONSAC [34].

Deep learning-based outlier rejection methods have grown lately, in which an initial correspondence set is first established by using a classic method (i.e., SIFT [18]) and an end-to-end fashion is used to determine the probability that each correspondence is an inlier. The LFGC [12] network is the first one to get rid of mismatches from the initial correspondence set by the deep learning-based manner. The network has used Multi-Layer Perceptrons (MLPs) and Context Normalization to cope with all the correspondences and performs well.

After that, some deep learning networks, such as LMR [32], ACNe [13], OANet [14], SCSA [16] and so on, are proposed to deal with the outlier rejection problem. Because LFGC [12] may fail to capture some correct correspondences in order to estimate the motion parameters, it is difficult to deal with some general matching problems, such as deformation and so on. Therefore, Ma et al. have presented a general framework to eliminate mismatches, named LMR [32], in which some images and geometric representations that are used to train. In ACNe [13], Sun et al. have put forward Attentive Context Normalization (ACN) and utilized it to capture and combine local and global contextual information. In OANet [14], Zhang et al. have come up with a Differentiable Pooling Layer and a Differentiable Unpooling Layer to work together to generate clusters and restore to the correspondences, in which the correspondences have been invariant to the input correspondence permutations. Meanwhile, the Order-Aware Filtering Block has been proposed to extract the global contextual information among the newly generated clusters. In SCSA [16], Liu et al. have introduced a spatial self-attention block to extract abundant contextual information among all the correspondences. Simultaneously, a channel self-attention module has been proposed to extract rich contextual information among all the channels. Afterwards, they combined both of them to improve the representation capability of potential correct matches.

However, the above methods fail to consider the relationship between each correspondence in the geometric local region, which cannot discriminately process potential inliers and potential outliers without ignoring the local structure. Therefore, a novel network with a stable local constraint (called LNCNet), i.e., local neighborhood correlation, is introduced, which can extract richer contextual information and obtain the feature map with the stronger presentation ability; therefore, it performs better in calculating the essential matrix and estimating the camera pose.

## 3. Method

In this section, we first formalize the problem in Section 3.1. After that, the local neighborhood correlation block and network architecture are described in Section 3.2 and Section 3.3, respectively. Finally, we introduce the loss function and implementation details in Section 3.4 and Section 3.5, respectively.

### 3.1. Problem Formulation

Our task aims to remove mismatches from the initial correspondence set, followed by essential matrix calculation and camera pose estimation. Firstly, the traditional SIFT [18] is used to find keypoints and corresponding descriptors of a given pair of images ($I,I^{\prime }$). Furthermore, then, an initial correspondence set $S=\left\{c_{1},c_{2},\ldots ,c_{N}\right\}\in {\mathbb{R}}^{N\times 4}$ is obtained according to a similarity constraint of descriptors, which consists of *N* initial correspondences. Furthermore, $c_{i}=\left(x_{i},y_{i},x_{i}^{\prime },y_{i}^{\prime }\right)$ is the *i* th initial correspondence, where ($x_{i},y_{i}$) and ($x_{i}^{\prime },y_{i}^{\prime }$) are the normalized coordinates of the correspondence under camera intrinsics and forced into the range [−1, 1]. We put the *S* set into our network, and we will obtain a corresponding probability set $w=\left\{w_{1},w_{2},\ldots ,w_{N}\right\}$, in which $w_{i}$ shows the probability of $c_{i}$ as an inlier and $w_{i}\in \left[0,1\right)$. Following LFGC [12], we choose the weighted 8-point algorithm to calculate the essential matrix $\hat{E}$, where the weighted 8-point algorithm merely focuses on inliers, so it is more robust than the 8-point algorithm. A series of operations can be formulated as:

(1) $$u=f_{\psi }\left(S\right)$$

(2) w=tanh(ReLU(u))

(3) $$\hat{E}=g\left(S,w\right)$$

where *u* is a set of logit values, each of tanh and ReLU is an activation function, $f_{\psi }\left(.\right)$ is our network function with related parameters ψ, and g(.) represents the weighted 8-point algorithm.

### 3.2. Local Neighborhood Correlation Block

In this section, the local neighborhood correlation block will be introduced in detail. To present the local neighborhood correlation constraint, building a local neighborhood structure of the initial matching set is necessary. Firstly, the initial correspondence set $S=\left\{c_{1},c_{2},\ldots ,c_{N}\right\}\in {\mathbb{R}}^{N\times 4}$ becomes the initial feature map set $F=\left\{f_{1},f_{2},\ldots ,f_{N}\right\}\in {\mathbb{R}}^{C\times N\times 1}$ through the multi-layer perceptrons. Secondly, we adopt the classic k-Nearest Neighbor (*KNN*) to find K neighbors with the shortest Euclidean distances to the initial feature map set $F=\left\{f_{1},f_{2},\ldots ,f_{N}\right\}\in {\mathbb{R}}^{C\times N\times 1}$ and construct a sketchy local neighborhood relationship. After that, we capture the neighborhood correlation between $f_{i}\in F$ and $f_{ij}$, termed LNC, where $f_{ij}$ is the *j*th neighbor in the local neighborhood of $f_{i}$. Finally, we filter and cluster the correspondence features according to the neighborhood correlation LNC.

**Build Local Neighborhood Correlation:**

To extract the local neighborhood related constraints, it is necessary to establish the local neighborhood structure of the initial feature map set $F\in {\mathbb{R}}^{C\times N\times 1}$. First of all, the classical KNN algorithm is used to choose the neighborhood $f_{ij}$ ($f_{ij}\in N_{f_{i}}$) to $f_{i}$ according to Euclidean distances. The *KNN* criterion can be defined as follows:

(4) $$w\left(f_{i},n_{j}\right)=\left\{\begin{array}{cc} 1, & n_{j}\in N_{f_{i}},\\ 0, & n_{j}\notin N_{f_{i}}. \end{array}\right.$$

where $w(f_{i},n_{j})$ is only a one-hot encoded weight and can roughly present the probability of $n_{j}$ being a neighbor of $f_{i}$; $f_{i}$ and $n_{j}$ are the selected initial feature map and the other initial feature map, respectively; $N_{f_{i}}$ is the *i*th element of the original local neighborhood feature map $N_{F}$.

**Figure 2:**
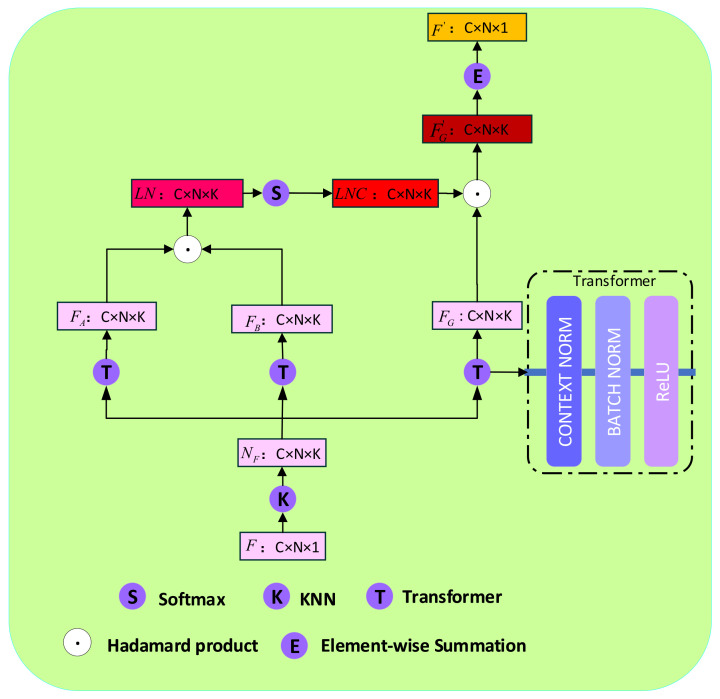
Local Neighborhood Correlation Block. First, the K-Nearest Neighbor (*KNN*) is used to divide the neighborhood roughly. Then, the local neighborhood correlation matrix LNC between the selected correspondence and any other correspondence in the local region is calculated, which is used to filter outliers, and finally, the new feature maps are aggregated.

The initial neighborhood feature map $N_{F}\in {\mathbb{R}}^{C\times N\times K}$ includes both inliers and outliers, so it is not accurate enough to estimate the essential matrix and camera pose. Hence, we construct the local neighborhood correlation matrix $LNC\in {\mathbb{R}}^{C\times N\times K}$ to alleviate this shortcoming, as described in Figure 2. To better capture the context information of each element in $N_{F}\in {\mathbb{R}}^{C\times N\times K}$, we map $N_{F}$ through two different transformers, each of which is composed of a Context Normalization layer, a Bath Normalization layer and a ReLU activation function, to gain two new features: $F_{A}\in {\mathbb{R}}^{C\times N\times K}$ and $F_{B}\in {\mathbb{R}}^{C\times N\times K}$. Through the hadamard product between $F_{A}\in {\mathbb{R}}^{C\times N\times K}$ and $F_{B}\in {\mathbb{R}}^{C\times N\times K}$, their neighborhood element similarity matrix $LN\in {\mathbb{R}}^{C\times N\times K}$ is obtained. The local neighborhood correlation matrix $LNC\in {\mathbb{R}}^{C\times N\times K}$ is obtained by softmax operation on the neighborhood element similarity matrix $LN\in {\mathbb{R}}^{C\times N\times K}$. The above series of operations can be recorded as:

(5) $$F_{A}=T\left(N_{F}\right)$$

(6) $$F_{B}=T\left(N_{F}\right)$$

(7) $$LN=H(F_{A},F_{B})$$

(8) LNC=Softmax(LN)

where $N_{F}\in {\mathbb{R}}^{C\times N\times K}$ denotes the initial neighborhood feature map; $F_{A}\in {\mathbb{R}}^{C\times N\times K}$ and $F_{B}\in {\mathbb{R}}^{C\times N\times K}$ are two new feature maps; $LN\in {\mathbb{R}}^{C\times N\times K}$ and $LNC\in {\mathbb{R}}^{C\times N\times K}$ are the neighborhood element similarity matrix and the local neighborhood correlation matrix, respectively; T(.), H(.) and Softmax(.) are the transformer, hadamard product and softmax operations, respectively.

**Local Feature Aggregation and Filter:**

**Figure 3:**
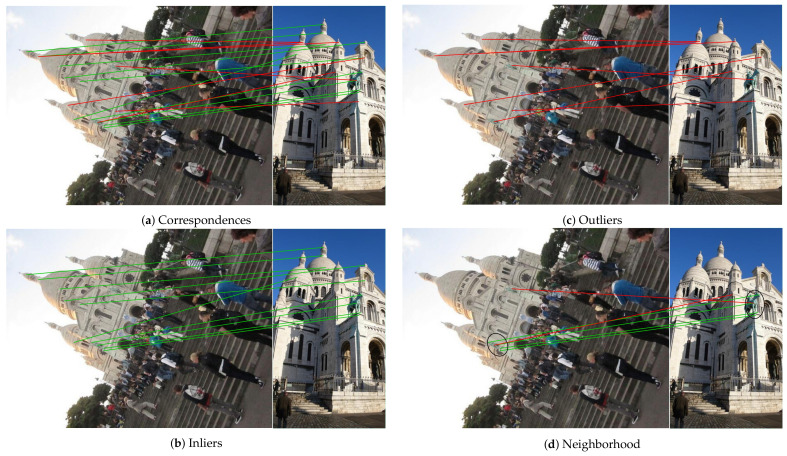
Diagram of local neighborhood correlation of correspondences. Inliers in the local neighborhood region have similar distribution, but outliers are randomly distributed.

According to the Bayesian principle [35], we know that correct correspondences have similar information, and correspondences with similar information are more likely to be inliers. Meanwhile, from Figure 3, we can find that these nearby inliers are distributed in a similar or identical spatial position and outliers are scattered in space. Because the outliers as noise will bring trouble to correspondences, it has a negative impact on calculating the essential matrix and estimating the camera pose. In particular, when there are outliers in the local neighborhood region, the new feature map of the selected correspondence clustering will achieve a bad performance, so filtering outliers in the local neighborhood region is required. That is to say, minimize the influence of the outliers on the selected correspondence and strengthen the support of inliers on the selected correspondence. We have obtained the local neighborhood correlation matrix $LNC\in {\mathbb{R}}^{C\times N\times K}$, which represents the similarity of each correspondence in the local neighborhood to the selected correspondence and use it to filter outliers.

First, we map the initial neighborhood feature map $N_{F}\in {\mathbb{R}}^{C\times N\times K}$ to the new feature map $F_{G}\in {\mathbb{R}}^{C\times N\times K}$ through the transformer. After that, we use the local neighborhood correlation matrix $LNC\in {\mathbb{R}}^{C\times N\times K}$ to filter the new feature map $F_{G}\in {\mathbb{R}}^{C\times N\times K}$ so that it can improve the probability of inliers as well as reduce the interference of outliers. These operations can be defined as:

(9) $$F_{G}=T\left(N_{F}\right)$$

(10) $$F_{G}^{\prime }=H\left(NFC,F_{G}\right)$$

where $F_{G}$ and $F_{G}^{\prime }$ are the feature maps before and after filtering, respectively; T(.) and H(.) are the transformer, hadamard product and softmax operations, respectively; $N_{F}$ and NFC are the initial neighborhood feature map and the local neighborhood correlation matrix, respectively.

The feature map $F_{G}^{\prime }$ includes more accurate neighborhood inlier information due to the filter operation. After that, we aggregate the neighborhood information on the selected correspondence, and the formula is as follows:

(11) $$F^{\prime }=E\left(F_{G}^{\prime }\right)$$

where E(.) is the element-wise summation operation; $F^{\prime }\in {\mathbb{R}}^{C\times N\times 1}$ is the output feature map.

Finally, the the output feature $F^{\prime }\in {\mathbb{R}}^{C\times N\times 1}$ is put into the rest of the architecture, as shown in Figure 1, to predict the probabilities of each correspondence in the initial correspondence set.

### 3.3. Network Architecture

From Figure 1, we can see the overall framework of LNCNet mainly consists of our proposed Local Neighborhood Correlation Block, PointCN Block, DiffPool&DiffUnpool Layer and Order-Aware Filtering Block. Firstly, we put our proposed Local Self-Attention Block at the front of the LNCNet so that it can provide more accurate information for the subsequent operations to improve the performance of the network. Secondly, a PointCN Block is made up of two identical contiguous modules, and each of them contains a Context Normalization layer, a Batch Normalization layer with a ReLU activation function and a Multi-Layer Perceptron. Finally, the DiffPool&DiffUnpool Layer consists of a Differentiable Pooling Layer, a Differentiable Unpooling Layer and three Order-Aware Filtering Blocks.

Inspired by OANet [14], we iteratively use sub-LNCNet twice, which is made up of the proposed Local Self-Attention Block 3 continuous PointCN Blocks, a DiffPool&DiffUnpool Layer, and the other 3 continuous PointCN Blocks in order. The initial correspondence set $S\in {\mathbb{R}}^{N\times 4}$ is put into the sub-LNCNet. Next, the outputs and their relevant residual information are put into the sub-LNCNet again. Finally, we carry out the ReLU and Tanh operations so that we can gain the weighted probability set $w\in {\mathbb{R}}^{N\times 1}$.

Compared with OANet [14], in our proposed LNCNet, *KNN* is used to coarsely divide the initial feature map set *F*. After that, the local neighborhood correlation matrix LNC between each selected correspondence and other correspondences in the neighborhood is calculated, which is used to filter outliers. Furthermore, we aggregate the more accurate neighborhood information to form a feature vector. Therefore, each of the feature vectors can embed more accurate and abundant information so that the proposed LNCNet performs outlier removal and camera pose estimation better.

### 3.4. Loss Function

We follow the idea of OANet [14], a hybrid loss function is adopted to guide LNCNet in training, including a classification loss and a regression loss. It can be formulated as:

(12) $$L=L_{c}\left(w,Y\right)+\lambda L_{r}\left(E,\hat{E}\right)$$

where λ is a parameter to obtain a trade-off between two losses. The first is the classification loss, where $L_{c}\left(.\right)$ is a binary cross-entropy loss. The ground-truth labels *Y* and the predicted probability set *w* are regarded as inputs. The weakly supervised labels *Y* can be calculated according to the essential matrix *E*, and the epipolar distance constraint [36] can be defined as:

(13) $$d\left(c,E\right)=\frac{{\left({p^{\prime }}^{T}Ep\right)}^{2}}{∥Ep{∥}_{\left[1\right]}^{2}+∥Ep{∥}_{\left[2\right]}^{2}+∥E^{T}p^{\prime }{∥}_{\left[1\right]}^{2}+∥E^{T}p^{\prime }{∥}_{\left[2\right]}^{2}}$$

where $c={\left(p^{T},{p^{\prime }}^{T}\right)}^{T}$ is an initial correspondence, and two keypoint positions are *p* and $p^{\prime }$. The *j*th entry of the vector *v* is $v_{\left[j\right]}$. If the geometric distance *d* is under the threshold ($10^{-4}$), the correspondence will be an inlier.

The second one is the regression matrix loss and can be written as:

(14) $$L_{r}\left(E,\hat{E}\right)=\sum_{i=1}^{N_{in}}\frac{{\left({p_{i}^{\prime }}^{T}\hat{E}p_{i}\right)}^{2}}{∥Ep_{i}{∥}_{\left[1\right]}^{2}+∥Ep_{i}{∥}_{\left[2\right]}^{2}+∥E^{T}p_{i}^{\prime }{∥}_{\left[1\right]}^{2}+∥E^{T}p_{i}^{\prime }{∥}_{\left[2\right]}^{2}}$$

where $\hat{E}$ is the essential matrix predicted by our network, and $N_{in}$ is the number of correct matches.

### 3.5. Implementation Details

The proposed LNCNet is shown in Figure 1, and its main parts were introduced in Section 3.3, each of which has 128 channels. The initial correspondence set $S\in {\mathbb{R}}^{N\times 4}$ (N=2000) is put into our proposed network. DiffPool&DiffUnpool Layer can map *N* matches to *M* clusters, where M=500. We gain the weighted probability set $w\in {\mathbb{R}}^{N\times 1}$ by ReLU and tanh operations. The whole network is implemented by Pytorch. According to experience, the learning rate of the Adam optimizer is $10^{-3}$. The iteration times are 500k, and the batchsize is 32. The weight parameter $L_{r}$ is initialized to 0, and after 20k iterations, we change it to 0.5.

## 4. Experiments

In the section, we first present datasets in Section 4.1. Second, we show evaluation metrics and comparative results in Section 4.2. Finally, we introduce ablation studies in Section 4.3.

### 4.1. Datasets

We choose Yahoo’s YFCC100M dataset [37] and SUN3D dataset [38] as the outdoor and indoor scene datasets, respectively.

**Outdoor Scenes:** Yahoo’s YFCC100M dataset [37] is as an outdoor scene dataset, which is made up of 100 million pieces of media data. We divide the media data into 71 image sequences, where 67 sequences are used to train networks and the remaining part as unknown datasets to test each network.

**Indoor Scenes:** We choose the SUN3D dataset [38] as an indoor scene dataset, which is a large-scale RGBD video dataset and can capture 3D information. We split the indoor scene dataset into 254 sequences, where 239 sequences are chosen to train networks. In addition, the remaining part of the above sequences are unknown scenes chosen to test all the networks. The indoor dataset is very challenging due to it with blurs and few distinctive features.

We test the robust and generalization abilities of each network in known and unknown scenes. Meanwhile, training sequences are split into disjoint subsets, i.e., the training set, the validation set and testing set are 60%, 20% and 20%, respectively. Of note, we use the results of testing in unknown scenarios as the main reference indexes, and the known scene results are just used as references.

### 4.2. Evaluation Metrics and Comparative Results

We show evaluation metrics and compare our proposed network (LNCNet) with other famous algorithms, i.e., RANSAC [7], LPM [10], PointNet++ [39], LMR [32], DFE [31], ACNe [13], LFGC [12], OANet [14] and their iterative variations (LFGC++ and OANet++) to accomplish outlier rejection and camera pose estimation tasks on indoor and outdoor datasets. The first two are classic traditional algorithms, whereas the rest are deep learning-based algorithms.

**Outlier Rejection:**Precision, Recall and *F*-score are regarded as evaluation metrics to evaluate the performance of some famous algorithms in outlier rejection. First, the definition of Precision(P) is the ratio between the number of positive samples and the number of predicted positive samples in the correspondence set. Second, the definition of Recall(R) is the ratio between the number of identified correct samples and the number of positive samples in the correspondence set. Finally, *F*-score(F) can be gained by 2*Precision*Recall/(Precision+Recall). The quantitative comparative experimental results are presented in Table 1. From that, we can find the performance of deep learning-based networks is much better than traditional RANSAC [7] on the two complex scenes. Because RANSAC [7] is fit for specific constraints and scenarios, it fails to perform good in the challenging datasets (the outlier ratio is often around 90%). However, deep learning-based networks are data-driven approaches, which have stronger abilities to reason and abstract the relationship among the correspondences. Therefore, they can obtain more accurate Precision, Recall and *F*-score values even from the putative correspondence set with vast scale outliers. Of note, our proposed network performs best in Precision, Recall and *F*-score on outdoor and indoor scenes on the whole.

**Figure 4:**
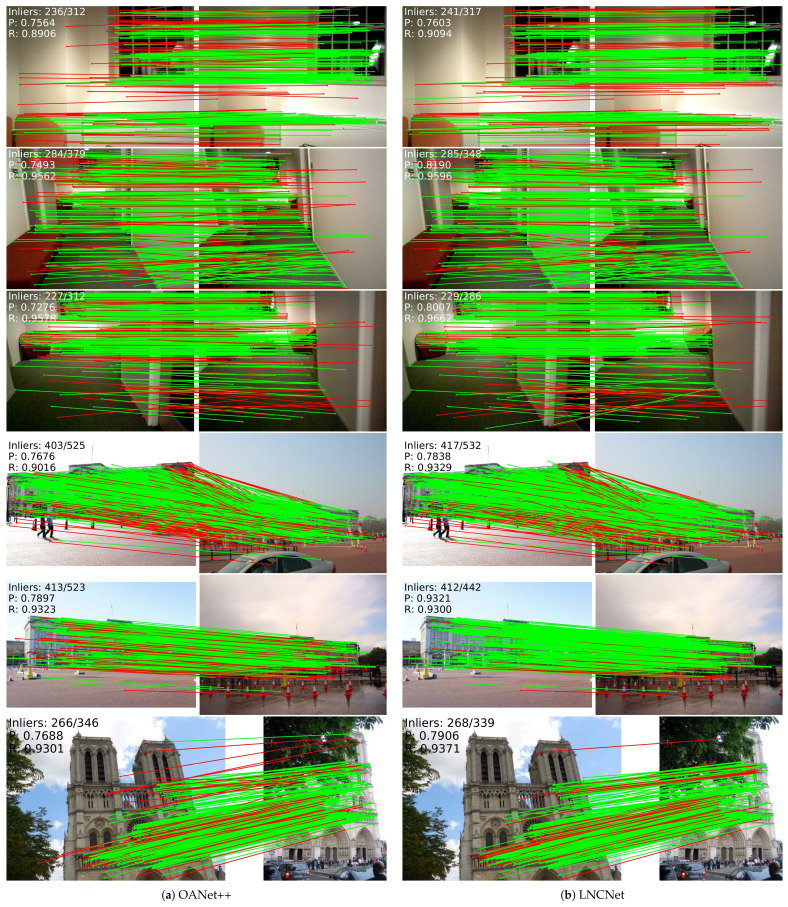
A part of the visualization results by (**a**) OANet++ (**left**) and (**b**) LNCNet (**right**). The top three images are the results of the SUN3D dataset, and the rest ones are the results of the YFCC100M dataset. Both of them are tested under the unknown scene.

Part of the visualization results are presented in Figure 4, where the left is OANet++, and our proposed network is on the right. The green line and the red line denote the right match and wrong match, respectively, and the information of inliers is clearly shown. In each set of pictures, our proposed network performs better than OANet++. Therefore, quantitative and partial visualization results can prove the effectiveness of LNCNet in outlier rejection well.

**Camera POse Estimation**: In this paper, we choose the mean average precision (mAP) of the angular differences under different error thresholds as evaluation metrics, where the angular difference is between the ground truth and the predicted vector for rotation and translation. Because mAP5° is more useful in the follow-up work, it is chosen as the default metric. Following OANet [14], RANSAC [7] with 0.001 threshold is as a post-processing step in the camera pose estimation. We test the general capabilities of networks in the unknown and known scenes for the camera pose estimation task in the two challenging datasets. From Table 2, we can find that our proposed network performs much better than other methods.

**Figure 5:**
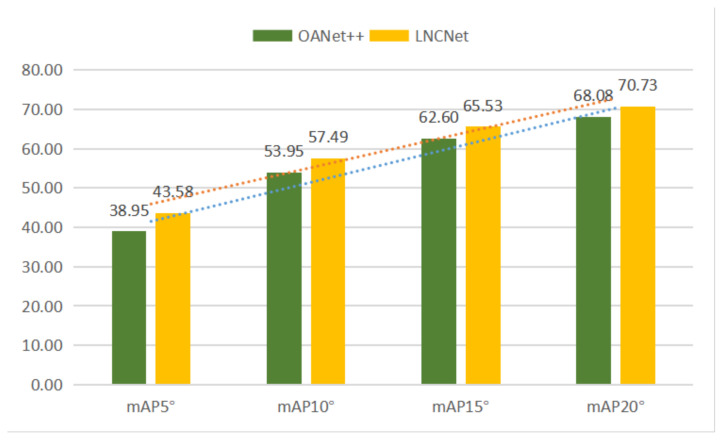
The results of OANet++ (green) and LNCNet (yellow) with the different mAP under the unknown YFCC100M scene without RANSAC.

**Figure 6:**
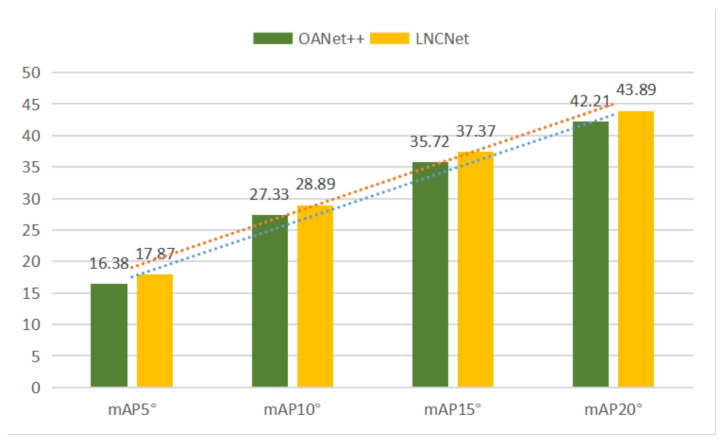
The results of OANet++ (green) and LNCNet (yellow) with the different mAP under the unknown SUN3D scene without RANSAC.

For indoor scenes, our network without RANSAC gains increases of 1.49% mAP5° and 2.30% mAP5° under unknown and known scenes compared to the second best network, respectively. Simultaneously, our network with RANSAC still performs better than other methods. For outdoor scenes, our network without RANSAC gets the mAP5° of 4.63% and 1.44% under unknown and known scenes compared to the second best network (OANet++), respectively. Meanwhile, increases of 2.65% mAP5° and 1.69% mAP5° are obtained under unknown and known scenes compared to OANet++ when using RANSAC. Figure 5 and Figure 6 show the performance of OANet++ and our proposed LNCNet with different error thresholds (i.e., mAP5°, mAP10°, mAP15° and mAP20°) on the YFCC100M dataset and SUN3D dataset, respectively. It proves that our proposed LNCNet performs better than OANet++ with different error thresholds under complex indoor and outdoor scenes. At the same time, it can be seen from the prediction lines (in Figure 5 and Figure 6) that the value of mAP increases linearly with the increase in the threshold.

### 4.3. Ablation Studies

**Figure 7:**
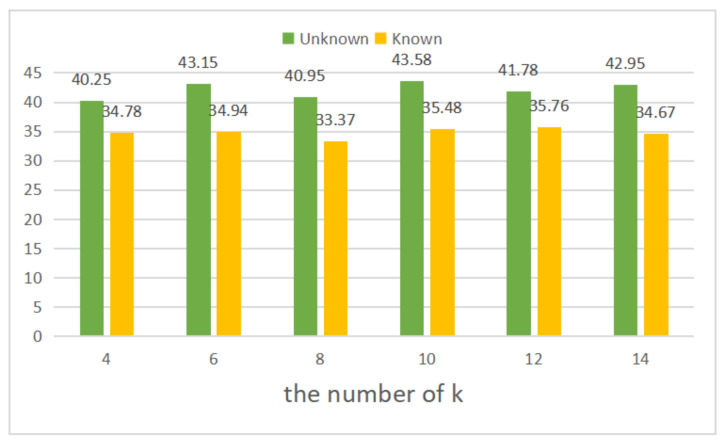
The results of LNCNet with different k under the unknown (green) and known (yellow) scenes without RANSAC.

In the section, we do the ablation study about how many neighbors we should choose in the LNCNet on the YFCC100M dataset. The performance of the proposed LNCNet with different *k*, e.g., $k=\left\{4,6,8,10,12,14\right\}$ is tested under unknown and known scenes. As shown in Figure 7, if the value of *k* is too large (14) or too small (4), the performance of our network will decrease. If the value of *k* is too small, we will fail to obtain enough neighborhood information. On the contrary, if the value of *k* is too large, many correspondences with less correlation may be divided into the neighborhood, which can decrease the performance of networks. Therefore, we select k=10 to determine the local region.

## 5. Discussions and Conclusions

In our work, the Local Neighborhood Correlation Network (LNCNet) is proposed to improve two-view correspondence learning. In particular, we fully utilize the local neighborhood correlation block so that we can gain the feature maps with stronger representation abilities among reliable correspondences in the local region. We tested our proposed LNCNet to accomplish the outlier rejection and camera pose estimation tasks under two complex datasets, and it performed better than other famous methods on the whole. However, because we use k-Nearest Neighbor (*KNN*) to roughly choose the local region, the time complexity may be a little high. Therefore, we plan to explore the variant version of *KNN* or other ways to solve the above problem in our future work. At the same time, we also plan to integrate information of different scales into our network so that our network can better complete the tasks of outlier removal and camera pose estimation.

## Figures and Tables

**Table 1:** Comparisons of outlier rejection under outdoor and indoor unknown scenes are shown in order. Bold indicates the best-valued index.

Algorithm	YFCC100M(%)			SUN3D(%)		
	P	R	F	P	R	F
RANSAC [7]	41.83	57.08	48.28	44.11	46.42	45.24
LPM [10]	43.75	65.65	51.72	44.28	55.42	50.63
PointNet++ [39]	48.42	61.16	54.05	45.64	83.43	59.00
DFE [31]	51.68	83.49	63.84	44.09	84.00	57.82
LMR [32]	50.73	66.12	55.19	44.88	58.21	52.71
ACNe [13]	54.56	86.92	67.04	46.44	84.23	59.87
LFGC [12]	53.12	85.51	65.53	47.24	83.45	60.32
LFGC++	53.71	85.57	66.00	45.82	**84.28**	59.36
OANet [14]	55.65	85.80	67.51	46.54	83.43	59.74
OANet++	54.55	**86.67**	66.96	46.95	83.77	60.17
LNCNet	**57.67**	86.21	**69.11**	**48.37**	83.49	**61.25**

**Table 2:** Comparisons of camera pose estimation under the outdoor and indoor scenes are reported in order. Results with and without RANSAC are also shown. Bold indicates the best-valued index.

Algorithm	YFCC100M (%)		SUN3D (%)	
	Known	Unknown	Known	Unknown
RANSAC [7]	5.82/-	9.08/-	4.38/-	2.86/-
PointNet++ [39]	34.69/11.49	45.85/15.75	21.00/11.80	18.79/10.29
DFE [31]	35.17/12.52	49.80/21.78	20.34/10.08	15.68/08.81
ACNe [13]	39.08/25.55	51.62/35.40	21.08/13.44	16.40/11.62
LFGC [12]	37.19/16.77	49.93/26.13	20.85/13.62	16.35/11.96
LFGC++	37.76/19.78	49.92/30.28	21.08/14.33	15.77/12.59
OANet [14]	41.40/31.00	51.45/35.07	22.29/19.22	16.95/13.69
OANet++	42.06/34.04	51.65/38.95	22.76/21.19	17.48/16.38
LNCNet	**43.75**/**35.48**	**54.30**/**43.58**	**23.05**/**23.49**	**18.00**/**17.87**

## Data Availability

MDPI Research Data Policies at the pubilcly available datasets of published papers [37] (link: https://dl.acm.org/doi/10.1145/2812802, accessed on 22 June 2021) and, Ref. [38] (link: https://ieeexplore.ieee.org/document/6751312, accessed on 22 June 2021). The images used in this paper are all from the above pubilcly available datasets.
